# Excessive and Unreliable Health Information and Its Predictability for Anxiety: A Cross-Sectional Observational Study

**DOI:** 10.7759/cureus.31247

**Published:** 2022-11-08

**Authors:** Puja Pallavi, Ajay K Bakhla, Ravi R Kisku, Rishi Guria, Mrityunjay Mundu, Rajni Bala

**Affiliations:** 1 Psychiatry, Rajendra Institute of Medical Sciences, Ranchi, IND; 2 Internal Medicine, Rajendra Institute of Medical Sciences, Ranchi, IND; 3 Surgery, Rajendra Institute of Medical Sciences, Ranchi, IND; 4 Psychology, Rajendra Institute of Medical Sciences, Ranchi, IND

**Keywords:** anxiety, unreliable health information, health anxiety, covid-19, information overload

## Abstract

Introduction: Being ignorant or unaware is not expected in a situation like the pandemic of COVID-19 with modern internet connectivity and the era of social media. However, information overload may itself lead to health anxiety.

Aims and objectives: This study investigated the predictability of health anxiety with information overload and sociodemographic profiles during the COVID-19 pandemic.

Materials and methods: A cross-sectional study was done among 400 caretakers of non-covid patients in a tertiary healthcare medical college. The consenting participants provided their sociodemographic details and responded to the short health anxiety inventory (SHAI), Beck anxiety inventory (BAI) and Information overload scale (IOS) for COVID-19.

Results: A total number of 400 participants aged 35.58 ± 10.57 years participated and out of which 88.2% acknowledged health-related anxiety and 56.8% for excessive use of social media. BAI measured anxiety was mild for 19.8%, moderate for 3.5% and severe for 3%. The linear regression analysis predicted health anxiety by three variables only: total anxiety as measured by the Beck anxiety inventory [β = 0.416, t = 9.318, p = 0.000], information overload (rejection of information) [β = 0.171, t = 3.126, p = 0.002], and excessive use of social media [β = 0.124, t = 2.888, p = 0.004].

Conclusion: Information overload, its rejection and excessive use of social media were found to be predictive of health-related anxiety.

## Introduction

The experience of having health-related concerns, along with their mental and bodily manifestations, may range from a continuum of normal concern to preoccupation with significant fear or interference with normal daily activities. The well-established mortality and morbidity of the COVID-19 pandemic indicate much higher psychological negative consequences [1]. COVID-19 is a health emergency that logically ignites health-related anxiety; however, many contributory factors may be responsible for its initiation and continuation, which may be diverse, including being health care professionals, risk of contracting a disease, social isolation, lower socioeconomic status [2], female gender, past or present psychiatric or comorbid chronic disease and urban habitat [3].

The role of perception and cognition may be crucial to the emotional reaction, which in turn influences knowledge and risk perception [4]. Despite the presence of reliable and authentic sources of information for COVID-19, most individuals have access to various internet and social media information, which remains the most common but, unfortunately, the most unreliable, emotionally charged, and anxiety-provoking sources of information for them [4,5]. However, the use of social media is not necessarily anxiogenic; a large quantitative study [6] found that the use of social media improves public health awareness and behavior for better protection against the COVID-19 pandemic. Yet another study found spending over two hours daily on COVID-19 news via social media is associated with anxiety [7]. However, the individual threshold of social media use duration may be debatable, and it may be identified as use, harmful use and dependence continuum. Harmful use may initiate anxiety and, on the other hand, anxiety itself, as cyberchondria leads to excessive COVID-19-related or any other health-related content searches on the internet. The significant amount of social media usage about health issues involves seeking information from various available sources in an attempt to gain accurate knowledge on the health-related issue or to counter the associated anxiety, but studies on the Zika virus also affirm that more accurate knowledge of the virus is associated with higher anxiety about the virus [8]. Excessive, inaccurate news and ambiguous information add up to many COVID-19-related adversities in the community, causing anxiety, stress responses and misplaced health-related behavior [5,9,10]. On the contrary, lower health anxiety is known for less protective health behavior [11]. The idea of measuring “information overload” and its impact started for medical issues with cancer [12] and was further studied with other health-related issues like sun-safe behavior [13], information on a healthy diet [14], atrial fibrillation [15] and recently adopted and validated for COVID-19 in Indian population [16].

We planned this study on the perception of information available to the general population by various means, in terms of excessive and unreliable health information and its predictability for health-related anxiety during the COVID-19 pandemic, which can be implicated or generalized for various other health-related information processing of public awareness provisions.

## Materials and methods

This study has been provisionally approved by the Institutional Ethics Committee, Rajendra Institute of Medical Sciences, Ranchi (no - 384/IEC/RIMS, dated October 30, 2021) and has therefore been conducted in accordance with the ethical standards laid down in the 1964 Declaration of Helsinki and its later amendments. This is a cross-sectional, survey-based observational study carried out during the post-COVID-19 pandemic in the months of October-November 2021. Convenient sampling using cross-sectional data collection from consenting participants by direct interview, with all efforts and intentions to not disclose the personal identity of any of the respondents. This is a cross-sectional, survey-based observational study carried out as per the Strengthening the Reporting of Observational Studies in Epidemiology (STROBE) Statement-Checklist for cross-sectional studies.

Study design

Selection and Description of Participants

A survey form including all sociodemographic information and selected scales and questionnaires was developed. Potential respondents were family members or caretakers of non-Covid patients visiting the medicine, psychiatry or surgery departments of a tertiary care hospital. Potential participants were asked about their willingness and consent to provide their basic information and reply to the survey questionnaire. It was recorded on the Google form by the interviewing investigator as a one-time cross-sectional participation only.

Sample size calculation [17] was calculated with recommended rules of thumb as events per variables (EPV) of 50 and the formula; n = 100 + 50i, where i refers to the number of independent variables in the model. We estimated five independent variables and calculated (n = 100+50*5) equals to 350.

Tools

*Sociodemographic Data Shee*t

Google survey form included questions and options of demographic details to be filled up by participants. The sociodemographic information included age, gender, education, religion, habitat, domicile state, socioeconomic status and marital status. The Questionnaire further included the history of past medical illness, existing physical illness, family history of psychiatric illness and type of employment.

Information Overload Scale (IOS) COVID-19

An original “Cancer Information Overload Scale” [12,16] was adopted and validated in India for use during the epidemic of COVID-19. It consisted of eight items with response provision on a 4-point Likert type scale from “strongly disagree=1,” “disagree=2,” “agree=3” to “strongly agree = 4.” It has shown good inter-item correlation, Cronbach alpha of 0.80, and acceptable convergent validity and discriminant validity. This scale has shown two factors: “Excessiveness of information”, consisting of five items, and “Rejection of information”, with three items.

Short Health Anxiety Inventory (SHAI)

This tool is comprised of 18-item questionnaires that measure health-related anxiety that includes worry about health, awareness of bodily sensations and fear of having an illness [18]. Each item of SHAI is scored with a four-point Likert scale of 0-3, with statements being rated as “I do not” (0) to “I spend most of my time” (3), resulting in a maximum aggregate score of 54. A cut-off score of 27 and above is considered problematic health anxiety [19]. Originally SHAI has shown a two-factor structure “fear of becoming ill - Illness Likelihood (IL)” and “fear of negative consequences of illness - Negative Consequences (NC).” The IL subscale contains items 1 to 14 and the NC subscale contains items 15 to 18. A systematic review and meta-analysis of SHAI [19] have found that it has acceptable Cronbach’s alpha scores, strong construct validity, and is sensitive to treatment across various studies.

Beck’s Anxiety Inventory (BAI)

It consists of 21 common somatic and cognitive symptoms of anxiety with a Likert scale scoring from 0 to 3 for each item. The highest possible score is 63, which may be classified as a score of up to 7 = minimal anxiety, 8 to 15 = mild anxiety, 16 to 25 = moderate anxiety and 26 to 63 = severe anxiety. The BAI [20] has been found to be an effective screening for anxiety with a good correlation between self-report and clinician rating. It has a reliability coefficient of 0.92 and test-retest reliability of 0.75.

Statistics

The data collected were entered into and analyzed with Statistical Packages for Social Science (SPSS)® (SPSS Inc, Chicago, IL, USA) version 16.0. Data normality was checked, and descriptive analysis for sociodemographic characteristics was done. Data collected as categorical variables were analyzed descriptively as percentage and frequency, whereas continuous data were described in mean and standard deviation. Pearson’s bivariate correlations were used to analyze the relationship between self-report measures like Short Health Anxiety Inventory - Illness Likelihood (SHAI-IL), Short Health Anxiety Inventory - Negative Consequences (SHAI-NC), Becks Anxiety Inventory (BAI), Information Overload - Excessiveness of Information (IO-EI) and Information Overload - Rejection of Information (IO-RI). Linear regression analysis assumptions such as linearity, normality, homoscedasticity and independence were considered, which partially supported the eligibility of the analysis. The presence or absence of health anxiety was taken as the dependent variable and other variables like age, gender, overall anxiety as measured by BAI, information overload (rejection), and “excessive use of social media” was taken as independent variables. We ran a multiple logistic regression analysis to evaluate variables that predicted health-related anxiety. The significance level was obtained with a p-value < 0.05 and a confidence interval (CI) of 95%.

## Results

Sociodemographic characteristics

A total of 400 patients were included in the study (response rate of 93.0%). Their mean age was 35.58 ± 10.57 years (range 17 to 77 years), and their mean years of education was 12.84 ± 2.56 years. Out of the total participants, about 238 (54.3%) were males, 217 (51.4%) were unmarried, 109 (45.6%) were between the ages of 35 and 64 years, 186 (46.5%) were Postgraduates and 282 (70.5%) were Hindu. The majority (85.2%) of participants came from urban habitat areas, with 64.5% from Jharkhand state, 71.2% from the middle socioeconomic class with 49.5% from regular employment. Only 72 (18%) had a past medical history, 75 (18.8%) with existing physical illness, and 52 (13%) had a family history of psychiatric illness. Additionally, 56.8% of participants agreed about excessive use of social media by themselves, and 88.2% of participants affirmed health-related anxiety. The severity of anxiety as measured by BAI was 19.8% mild, 3.5% moderate and 3% severe anxiety (Table 1).

**Table 1:** Sociodemographic characteristics (N=400)

	Mean ± SD	Min	Max
Age in years	35.58 ± 10.57	17	77
Education in years	14.15 ± 3.88	5	20
Information Overload score	21.76 ± 4.24	8	32
		n	%
Gender	Male	238	54.3
	Female	162	45.7
Marital Status	Unmarried	217	51.4
	Married	205	48.6
Education	Post Graduate	186	46.5
	Graduate	163	40.8
	Others	51	12.8
Religion	Hindu	282	70.5
	Muslim	15	3.8
	Others	103	25.8
Habitat	Urban	341	85.2
	Suburban	46	11.6
	Others	13	3.2
State	Jharkhand	258	64.5
	Out of JKD	142	35.5
Socioeconomic status	Lower Middle	102	25.5
	Middle	285	71.2
	Upper	13	3.2
Type of Employment	Regular	198	49.5
	Contractual	60	15
	Others	142	35.5
Domain of Employment	Government	162	42.2
	Private	146	36.5
	Others	85	21.2
Past Medical History	No	328	82
	Yes	72	18
Existing Physical Illness	No	325	81.2
	Yes	75	18.8
Family History of Psychiatric Illness	No	348	87
	Yes	52	13
Social media excessive use	Agree	227	56.8
	Disagree	173	43.2
HAI (mean 37.87±9.12)	No	47	11.8
	Yes	353	88.2
BAI (26.2%)	Mild	79	19.8
	Moderate	14	3.5
	Severe	12	3.0

Relationship of health anxiety with information overload

Correlation analysis was performed among self-report measures of health anxiety subscales, anxiety as measured by BAI, Information subscales and total scores, as well as age and years of education (Table 2). All self-report measures significantly and positively correlated among themselves with < 0.01 significance level, except < 0.05 level significance between SHAI subscale SHAI-NC and BAI total score. Age was negatively and significantly correlated with both the BAI total score and SHAI total score (p < 0.01) and SHAI subscale SHAI-NC and IC (p < 0.05). It was also found that age was not associated with information overload in the total score or its subscales. A statistically non-significant negative correlation was found between years of education and information overload total score, and both subscales and BAI-T (Table 2).

**Table 2:** Inter-correlations among self-report measures Correlation is significant at the ∗p-value < 0.05; ∗∗p-value < 0.01 (2-tailed). Notes: BAI-T = Beck Anxiety Inventory – Total score; IO-EI = Information Overload “Excessiveness of Information”; IO-RI = Information Overload “Rejection of Information”; SHAI-NC = Short Health Anxiety Inventory NC; SHAI- IL= Short Health Anxiety Inventory IL

	Age	Education in Years	BAI - T	SHAI-NC	SHAI-IL	HAI total scores	IO1	IO2	IOS total
Age	1								
Education in Years	0.05	1							
BAI-T	-0.16^**^	-0.02	1						
SHAI-NC	-0.10^*^	-0.01	0.11^*^	1					
SHAI-IL	-0.12^*^	0.02	0.53^**^	0.36^**^	1				
HAI total scores	-0.13^**^	0.01	0.49^**^	0.59^**^	0.96^**^	1			
IO-EI	0.03	-0.04	0.17^**^	0.24^**^	0.21^**^	0.25^**^	1		
IO-RI	0.06	-0.03	0.22^**^	0.27^**^	0.27^**^	0.31^**^	0.62^**^	1	
IOS total	0.05	-0.04	0.21^**^	0.27^**^	0.26^**^	0.30^**^	0.94^**^	0.84^**^	1

Prediction analysis

Among the sociodemographic variables, age, gender and excessive social media use were included in the regression analyses. Other variables like past or family medical history were not included as they lacked any significant correlation with the criteria. (Table 3) The linear regression analysis predicted health anxiety by three variables only: total anxiety as measured by the Beck anxiety inventory, information overload (rejection of information) and excessive use of social media. Among these three, Anxiety, as measured by BAI-T [β = 0.416, t = 9.318, p = <0.001] was found to be the most significant, as expected. However, information overload (rejection of information) [β = 0.171, t = 3.126, p = 0.002], and excessive use of social media [β = 0.124, t = 2.888, p = 0.004] were found. A significant regression equation was found (F (6,393) = 29.436, p=<0.001 with an R2 of 0.310). Younger age and female gender (gender was coded 1 for males and 2 for females) could not predict health anxiety significantly.

**Table 3:** Logistic regression analysis on factors significantly associated with health anxiety Dependent Variable: HAI total scores, Constant B = 27.721, SD = 2.811, t = 9.861 (p-value = <0.001). *R = 0.557, R2 = 0.310, (change statistical significance p=.000). Excluded variable-IOS Total. Notes: BAI-T = Beck Anxiety Inventory – Total score; IO-EI = Information Overload “Excessiveness of Information”; IO-RI = Information Overload “Rejection of Information”; Excessive SMU=Excessive Social Media Use.

Predictors	Estimate B	Std. Error	Beta	t	P-value
BAI-T	0.56	0.06	0.42	9.32	<0.001***
IO-EI	0.18	0.17	0.06	1.11	0.269
IO-RI	0.89	0.28	0.17	3.13	0.002**
Age	-0.08	0.04	-0.09	-2.20	0.028
Gender	-0.38	0.83	-0.02	-0.46	0.643
Excessive SMU	2.32	0.80	0.12	2.89	0.004**

## Discussion

The present study evaluated health anxiety, anxiety and information overload, immediately after the COVID-19 pandemic and the specific impact of anxiety and information overload on health anxiety. In the current study, the results confirmed the significant impact of anxiety and the “rejection” components of Information overload and excessive use of social media use on health anxiety.

Health anxiety, in its severe presentation, was used to describe hypochondriasis in the Diagnostic and Statistical Manual of Mental Disorders (DSM) - IV and is currently known as somatic symptom disorder or illness anxiety disorder. Anxiety has been very extensively studied during this COVID-19 pandemic, but health anxiety specifically has not received much attention as there are overlapping concepts under the broad understanding of anxiety. The prevalence of health anxiety in the general population is approximately 5.7% [21], but in a large clinical sample, about 20% of patients were found with health anxiety, as measured by Health Anxiety Inventory [22] and studies reported increasing prevalence of health anxiety even before COVID-19 onset [23]. However, the COVID-19 pandemic is a situation that provokes unprecedented health-related anxiety and other psychosocial reactions [24]. This justifies the very high cross-sectional health anxiety symptoms of 88.2% among the general population found in our study. However, the diagnosis of health anxiety disorder needs a duration criterion of six months. In a similar study measuring health anxiety, we found a mean SHAI score of 15.1 ± 7.0 in Turkey [3] or (14.68 ± 6.58) in Germany [25]. These are low in comparison to the 37.87 ± 9.12 of our study, and the difference may be attributed to different cultural variables in experiencing and expressing anxiety in addition to different phases of the COVID-19 pandemic.

We measured anxiety with BAI, which is a very well-established rating scale for anxiety, whereas SHAI is a specific health anxiety measurement. Significant positive correlation between BAI and SHAI (r=.492; p-value < 0.01) indicates that they were sensitive to change together in similar directions and to affirm good convergent validity for each other.

Similarly, both subscales and total scores of the IOS showed a significant positive correlation with the SHAI total score (r=0.30, p=<0.01) and with BAI (r=0.21, p=<0.01). However, logistic regression affirms the predictability of health anxiety by anxiety (as measured by BAI) and the “information rejection” factor of information overload. Our study affirms the role of information overload on health anxiety significantly even when a significant association could not be established with other previously found psychosocial attributes like female gender, education, lower socioeconomic status, urban habitat and past or present psychiatric or comorbid chronic disease [2,3].

Our study is in concordance with a few recent studies that used validated measures of information overload for the COVID-19 pandemic with anxiety, depression, post-traumatic stress disorder [26], cyber aggression, Confucian responsibility thinking, depression and anxiety [27]. A similar construct was used by a German study [25], they found health anxiety was correlated positively with cyberchondria and maladaptive emotion regulation but negatively correlated with the perception of being well-informed and adaptive emotion regulation. Adaptive and maladaptive emotion regulation may be the key cognitive difference, but the subjective perception of being informed (rated 0-100) is closer to the idea of “information satisfaction.” This satisfaction should be the opposite of “rejecting information” but not necessarily “excessiveness of information.” This can be considered to be of speculative concordance with the main finding of a significant positive correlation between health anxiety and rejecting information but not with information overload in our study.

We also found that subjective self-perceived excessive use of social media significantly predicts health anxiety. This finding is in accordance with reports of excessive social media use [11], which may be attributed to the implementation of lockdown, self-isolation and self-quarantine rules, and social distancing in a country. Additionally, there is misuse, spreading fake news and information on social media about COVID-19 [28], which all contribute to anxiety and health anxiety. A recent meta-analysis concluded that fear of COVID-19 was strongly related to anxiety [29].

Our study found that unreliable information and rejection of information, significantly predict health anxiety in comparison to excessive components of information overload. Information directly depends on the availability or public access to a pandemic or illness-related sources of information and is least guided by its reliability. However, the present study has a few limitations, like it was only conducted in one city, has a cross-sectional design and might be missing other contributing factors for anxiety. However, the sample of this study consists of a highly educated class (87% above graduation), but actual literacy in India is overall low. The easy, low cost and round-the-clock available internet-based social media and television remained the main source of information and misinformation even among healthcare professionals [30]. Many contradictions, rapidly changing information and fake news took away its reliability and indeed propagated stigma. Suggested preventive strategies may include strict information policy during pandemics or other public health situations. This policy must inculcate a strong and effective health information system by the administration or government. There is a need to quickly identify and de-legitimize the sources of fake and unscientific information [28]. There should be promoted publicity of reliable and scientific internet sources, media reporting guidelines and education about media use.

There are certain limitations of the study; this study used convenient sampling which limits its generalizability. It was conducted among subjects in proximity to patients and the hospital environment, usually educated, using social media like WhatsApp, Facebook, etc. Future studies may be planned with a better methodology for other common illnesses, their information impact, and related health anxiety.

## Conclusions

In this study, 88.2% of participant affirms health-related anxiety during the COVID-19 pandemic, we found three predictors of health anxiety namely total anxiety as measured by the Beck anxiety inventory, information overload (rejection of information), and excessive use of social media. The finding affirms the fact that overall general anxiety and “health-anxiety” are often co-occurring. This study highlights the anxiogenic role of excessive health information with excessive use of social media. The information overload consists of excessiveness which may be unreliable, unscientific, false treatment claims, or fake stories, resulting in “rejection of information,” which has much significant predictability of health anxiety and its consequences. These findings emphasize the provision of reliable and scientifically valid and adequate information dissemination with all forms of media and more specifically social media for the prevention of “information induced health-anxiety.”
